# Policy analysis of the global financing facility in Uganda

**DOI:** 10.1080/16549716.2024.2336310

**Published:** 2024-06-19

**Authors:** Phillip Wanduru, Doris Kwesiga, Mary Kinney, Asha George, Peter Waiswa

**Affiliations:** a School of Public Health, Makerere University College of Health Sciences, Kampala, Uganda; b Department of Global Public Health, Karolinska Institutet, Stockholm, Sweden; c School of Public Health, Faculty of Community and Health Sciences, University of the Western Cape, Cape Town, Bellville, South Africa

**Keywords:** Global Financing Facility for Women, Children and Adolescents: Examining National Priorities, Processes and Investments, Uganda, global financing facility, global health initiative, policy analysis, RMNCAH (Reproductive, Maternal, Newborn, Child, and Adolescent Health)

## Abstract

**Background:**

In 2015, Uganda joined the Global Financing Facility (GFF), a Global Health Initiative for Reproductive, Maternal, Newborn, Child, and Adolescent Health (RMNCAH). Similar initiatives have been found to be powerful entities influencing national policy and priorities in Uganda, but few independent studies have assessed the GFF.

**Objective:**

To understand the policy process and contextual factors in Uganda that influenced the content of the GFF policy documents (Investment Case and Project Appraisal).

**Methods:**

We conducted a qualitative policy analysis. The data collection included a document review of national RMNCAH policy documents and key informant interviews with national stakeholders involved in the development process of GFF policy documents (*N* = 16). Data were analyzed thematically using the health policy triangle.

**Results:**

The process of developing the GFF documents unfolded rapidly with a strong country-led approach by the government. Work commenced in late 2015; the Investment Case was published in April 2016 and the Project Appraisal Document was completed and presented two months later. The process was steered by technocrats from government agencies, donor agencies, academics and selected civil society organisations, along with the involvement of political figures. The Ministry of Health was at the center of coordinating the process and navigating the contestations between technical priorities and political motivations. Although civil society organisations took part in the process, there were concerns that some were excluded.

**Conclusion:**

The learnings from this study provide insights into the translation of globally conceived health initiatives at country level, highlighting enablers and challenges. The study shows the challenges of trying to have a ‘country-led’ initiative, as such initiatives can still be heavily influenced by ‘elites’. Given the diversity of actors with varying interests, achieving representation of key actors, particularly those from underserved groups, can be difficult and may necessitate investing further time and resources in their engagement.

## Paper context

- **Main findings:** Existing policy priorities and platforms helped to shape the Global Financing Facility agenda in Uganda, with leadership from the Ministry of Health and subtle yet clear influences from global actors and national civil society.
- **Added knowledge:** The study contributes to the understanding of the complexities in translating a globally conceived initiative into one that is locally owned.
- **Global health impact for policy and action:** Global health initiatives must work with government to enable local ownership thorough more engagement with diverse national stakeholders in order to understand their expectations from inception and address them as part of processes of strengthening robust policy review and implementation.

## Background

The Global Financing Facility (GFF) was introduced in 2015 as a global financing model aimed at supporting countries with the greatest needs in women’s, children’s and adolescents’ health [1]. The Global Financing Facility is a multi-donor trust fund housed within the World Bank’s organisational framework and Uganda was one of the first partner countries identified in 2015 [2]. Uganda has made significant progress in Reproductive, Maternal, Newborn, Child and Adolescent Health (RMNCAH)). Nonetheless, it remains with high burdens of maternal and child mortality, requiring additional global resources to support the relevant programmes [3–5].

The GFF is a welcome initiative for RMNCAH, with a lot of promise like similar global health initiatives, such as Global Fund for HIV, Tuberculous and Malaria, the U.S. President’s Emergency Plan for AIDS Relief, and The Global Alliance for Vaccines and Immunization. Scholars in Uganda have been studying global health initiatives and regard them as powerful entities that significantly influence national health policy and prioritisation [6–8]. Since global health initiatives are typically conceived globally, implementation and adaptation of their ideas often diverge from the initial plan due to complex political and contextual realities at the national level [9]. This external influence typically operates in collaboration with that of in-country technical experts and politicians [10,11]. Rather than imposing predefined priorities, the GFF assists countries in identifying and addressing their own RMNCAH priorities through the development of an Investment Case (IC), a national policy document outlining these priorities, presenting a plan and accompanying budget. The GFF then provides a grant tied to the World Bank loan, which is meant to support the implementation of the IC and is described in the World Bank’s Project Appraisal Document (PAD) for the specific loan [12]. The GFF grant is routed through the World Bank, which handles disbursement and management.

The GFF arrangement provides a unique avenue for countries to achieve RMNCAH outcomes in the Sustainable Development Goal context. To date, there have been very few independent studies assessing the GFF policy processes and operational mechanisms. Some studies posit that the GFF might be the same top-down approach as some other global health initiatives, with limited country leadership and alignment to country systems [13–15]. An evaluation of the GFF implementation process in Uganda found some progress on several key indicators along the RMNCAH continuum of care. However, this was inadequate to reach nationally set targets. There were also implementation challenges, despite receipt of additional funds from GFF.

The evaluation report indicated that this was ‘a learning and foundational phase’ for the GFF, with the hope that lessons could be drawn from what happened in order to inform the next phase and future implementation [16]. While the evaluation examined implementation progress, more needs to be understood about the complex realities of the policy processes related to the introduction of the GFF in Uganda.

To support this learning, this study set out to understand the policy process of the GFF initiation in Uganda with a specific focus on the development of the two main GFF policy documents, that is, the IC and PAD. The study takes the actors into account as well as the context, governance structures, official and unofficial processes. It aims to further inform the process of initiating global health initiatives in countries and specifically support learning around the GFF processes.

## Methods

### Study design

This was a descriptive, exploratory, qualitative case study. It retrospectively focused on the period 2015–2016 when the GFF was introduced. A case study methodology was applied in order to explore and understand the policy process in round one of the GFF at national level [17]. This study focused on development of the two GFF planning documents (IC and PAD). This paper refers to them as ‘GFF policy documents’ because they are foundational to their national operations, although they were not created solely by the GFF.

The study was done as part of the ‘Countdown GFF policy analysis collaboration,’ a multidisciplinary group of academics and partners working in the African region [12]. The study applied an adapted framework based on the health policy triangle [18].

### Study setting

Uganda has achieved substantial progress on RMNCAH in the past few decades. Nonetheless, related mortality remains unacceptably high, with low effective coverage across the continuum of care for RMNCAH [16]. The GFF documents were developed around the time when Uganda was holding presidential and parliamentary elections. Despite the elections, there were few changes in political leadership, with the president remaining in office and his ruling party gaining many parliamentary seats. As a result, the same ruling party retained significant influence over various national-level priorities and decisions. Furthermore, although the health minister who was in charge of overseeing the GFF changed around this time, the successor was a member of the ministry’s top leadership, allowing for seamless process continuity. See supplementary file 1 for details on the country context.

### Data collection and management

Data collection comprised a document review and key informant interviews (Supplementary file 2). The document review included eight relevant national policy documents from the time period 2015–2016 (Table 1) and was geared toward gaining a thorough understanding of the RMNCAH policy landscape at the time of the GFF document development. These documents were selected because they were national-level policy documents that were in effect when the GFF documents were developed.

**Table 1.** List of documents reviewed.

Name of document	Duration	Government agency that designed policy
National Health policy (MOH Uganda 2010)	2010/11-2019/20	Ministry of Health
National Development Plan (NPA 2010)	2010/11–2014/15	National Planning Authority
Health Sector Strategic and Investment Plan (HSSIP) (MoH 2010)	2010/11–2014/15	Ministry of Health
Second National Development Plan (NDPII) (Finance 2015)	2015/16–2019/20	National Planning Authority
Report of the committee of national economy on the request by government to borrow SDR 78.5 MILLION (US 110.0 million equivalent) form the international development association (IDA) of the world bank group to support the Uganda reproductive maternal and child health(Parliament of Uganda 2016)	N/A	Parliament of Uganda
The Republic of Uganda. Investment case: Reproductive, Maternal, Newborn, Child and Adolescent Health (RMNCAH) sharpened plan of Uganda 2019/2020. (Ministry of Health 2016)	2016/17–2019/20	Ministry of Health
A promise renewed reproductive maternal, newborn, and child health sharpened plan for Uganda 2013(MOH Uganda 2013)	2013/14–2016/17	Ministry of Health
MOH. HEALTH SECTOR DEVELOPMENT PLAN [Internet]. 2015. (MOH 2015)	2015/16–2019/20	Ministry of Health

Key informant interviews were purposively conducted with respondents who had been involved in the development of the GFF policy documents. The aim was to gain deeper perspectives into the process and why specific content was included in the policy documents. A total of 22 key informants were contacted and 16 were interviewed (Table 2). They included individuals from the government (the Ministry of Health, donors (World Bank, GFF, multi-laterals and bilaterals), Civil Society Organizations (CSOs), and academia. All interviews were conducted in English, either face-to-face or remotely (on Zoom or by telephone). The average interview length was 40–60 min. Document review and data collection activities were conducted between October 2022 and February 2023.

**Table 2.** Key informants by organisation.

Type of respondent	Total contacted	Interviewed
Government	6	5
Donor (WB, bilateral, multilaterals)	8	4
Other actors (CSOs and academia)	8	7
Total	22	16

Following the completion of our data collection and preliminary analysis, we presented the study findings to stakeholders in two validation meetings. Participants of the meetings included representatives from Ministry of Health (MoH), the GFF secretariat in Uganda and members of CSOs. From these meetings, we were able to validate the findings and also to get further insights that further strengthened our data.

### Data analysis

The research team met weekly for one hour from October 2022 to March 2023 to co-develop the data collection and analysis tools (Supplementary File 3). We applied a two-pronged approach to analysis. For the document review, we conducted a content analysis of the documents, systematically analyzing the manifest contents of the documents guided by predefined themes in the framework.

For the interviews, we used thematic analysis but were also guided by the study framework. Coding was done by two individuals from the research team separately (DK and PhW). It took an iterative approach, with some additional codes added as new themes emerged. After generating initial codes, we also held multiple meetings with the Countdown GFF analysis collaborative to reflect on the analysis and develop our final themes. Both document reviews and interviews were coded in NVivo software.

### Positionality, reflexivity and ethics consideration

This study was approved by the research and ethics committee at Uganda Christian University (Reference number: SPH-2022-316). All participants provided informed verbal or written consent and were assured of confidentiality of their responses, including anonymity during dissemination of findings.

Measures were taken to ensure rigor of the case study approach, such as engagement with stakeholders before data collection, voluntary participation of participants, seeking peer and expert feedback, audit trail with clear mapping of the research process and triangulation of data sources. Feedback sessions took place with the Ugandan Ministry of Health and the GFF secretariat for validation of results.

Supplementary file 4 provides additional details on the Countdown GFF policy analysis collaboration equitable partnership agreement and author positionality. The senior author (PW) is a key expert on RMNCAH in the country, serving on government committees and he led the end-line evaluation of the first phase of the GFF in Uganda. His insider position facilitated the team’s access to key respondents and the selection of relevant policy documents. However, PW did not participate in interviewing or coding and this allowed for more independent perspectives to emerge. AG also serves on the GFF Results Advisory Group and while this gives her further insight into GFF aims, her role is independent of the GFF.

## Results

The results are presented according to three themes drawn from the analysis, including the descriptive elements of the policy process, factors that influenced the process and perceptions of stakeholders involved. We considered both formal and informal processes that influenced the development of these documents.

### Theme one: policy timelines alignment, and content

#### Policy timelines

Uganda commenced work on the GFF towards the end of 2015 [19]. By April 2016, the Investment Case was published, under the title ‘Investment Case for Reproductive, Maternal, Newborn, Child and Adolescent Health (RMNCAH) Sharpened Plan Uganda’ (2016/17–2019/20) [20]. Two months later, in July 2016, the Project Appraisal Document was published and presented to the World Bank board, designated as the ‘Uganda Reproductive, Maternal, and Child Health Services Improvement Project’. It detailed the specific investments that would be supported by the GFF fund and the International Development Association credit [19,21]. By December 2016, the International Development Association loan proposal was formally tabled for approval by the Parliament of Uganda [22].

Respondents partly linked the rapid policy development to an already existing promise renewed policy that was simply adopted.

Unlike other policies, the Investment Case was not difficult to create. We already had the Promise Renewed which had been developed comprehensively through a thorough consultative process. It was adopted in the creation of an Investment Case. (Respondent 7)

#### Policy alignment

**Figure 1.**
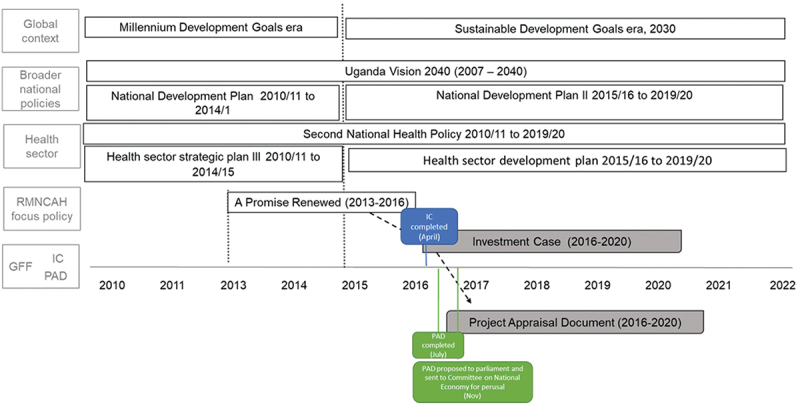
Policy and process timeline relating to the GFF country documents.

As mentioned, the Investment Case was adapted from Uganda’s key RMNCAH implementation guidance policy document, ‘A Promise Renewed: Reproductive Maternal, Newborn and Child Health Sharpened Plan for Uganda’ (2013–2017) [23]. The Promise Renewed was itself drawn from a larger ‘Roadmap for Accelerating the Reduction of Maternal and Neonatal Mortality and Morbidity in Uganda,’ which ran from 2007 to 2015 [24]. These RMNCAH policy documents aligned with their corresponding national and global development policies (Figure 1).

#### Content of policy documents

Our comparison of the Promise Renewed, Investment Case and Project Appraisal Document confirmed mostly overlapping core concepts and priorities but some important distinctions (Table 3 and Supplementary File 5). Both the ‘Promise Renewed’ and IC delineated five strategic shifts that serve as basic principles for achieving the ambitious goals of RMNCAH implementation. These shifts included: i) emphasising evidence-based high-impact solutions, ii) increasing access for high-burden populations such as adolescents, iii) geographic focusing, which includes prioritising districts with more preventable deaths, iv) addressing the broader multi-sector contextfor example, working with the education sector and v) ensuring mutual accountability. However, a significant change was the emphasis on adolescent health plus civil registration and vital statistics in the IC, which was not the case for ‘A Promise Renewed’.

**Table 3.** Mapping of content in three relate policy documents.

	Promise Renewed (2013)	Investment Case (April 2016)	Project Appraisal Document (June 2016)
Type of document	National level guide	National level guide	World Bank project document
Focus areas	Maternal, newborn, infant, and child health services	Maternal, newborn, infant and child health, plus adolescent health.	Improving health service delivery for RMNCAH in targeted districts and strengthening birth and death registration services
Value of budget/investment	USD 681 million USD budget	Three scenarios budgeted 1. US$ 1.6B if current coverage maintained 2. US$ 1.92B with rapid scale up of prioritised core and expanded package 3. US$ 2.2B if expanded package delivered nationwide	USD 140 million
Description of funding allocation	Non-specific: funds allocated in broad packages	Non-specific: funds allocated in broad packages	Specific/clear: four components described with allocated resources per component
Length/duration of investment	5 years (2013 to 2017)	5 years (2016/17–2019/20)	5 years (2017–2021)

The content of the IC and PAD aligned addressing RMNCAH through a holistic approach, focusing on improving service delivery along the continuum of RMNCAH care and health system aspects, with a particular emphasis on human resources (specifically midwives), scaling up results-based financing mechanisms, and improving care quality. Civil Registration and Vital Statistics was a key component within both documents. This has links to the Maternal Perinatal Death Surveillance and Response programme and birth registration.

### Theme two: factors influencing the content of the policy documents

The policy content of the GFF documents was broadly shaped by the use of evidence and the influence of technical and political actors. All respondents shared that data and evidence were the basis of informing prioritisation processes for the IC. This was also reported to be part of typical policy development processes that require a policy to be evidence-based. A variety of tools were used, including the Lives Saved Tool, Bottleneck Analysis and costing tools to identify context-relevant, high-impact interventions and cost-friendly priorities.

Data was key in informing our prioritization as you may know. We had costing data, we also looked at the highest-impact interventions. We were trying to understand which interventions require the least cost and yet have a high impact. (Respondent 1)

**Figure 2.**
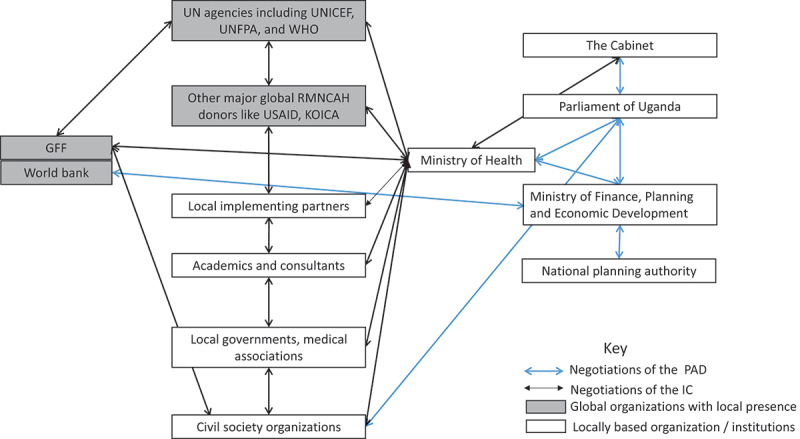
Interactions between actors in the development process of the GFF policy documents.

Various actors played key roles in developing and approving the GFF policy documents, influenced both by their specific involvement in the process, as well as by the power they held. Figure 2 maps the interactions between the different actors in the development of the IC and PAD. For the IC, the Ministry of Health served as a core and central actor in its development and took the lead in driving this participatory and country-led process. Indeed, at the initiation of the GFF process, Ministry of Health brought together a diverse group of stakeholders to develop the IC. This process was facilitated by an existing RMNCAH Technical Working Group, whose membership included organisations that had both global and local offices, such as UNICEF, UNFPA and WHO. In addition, there were representatives from district health authorities, academia, Civil Society Organizations and other RMNCAH implementing partners.

It is important to note that even before GFF, the MoH was already working with a well-established RMNCH technical working group to develop policies. The GFF required that a country platform be set up for the development of the IC, but the Ugandan team preferred to use the existing technical working group structure, whose composition was eventually broadened to be more inclusive as the extended MCH technical working group, as explained by respondents 1, 7 and 16. This expansion saw a more deliberate representation of academics and CSOs who were invited to participate in that group.

Like they [GFF] said, every country must establish what they called a country platform. They should have a new structure for implementing the in-country activities. But Uganda said no, for us we cannot. We have already our structures which we are using so we cannot have a parallel structure. Then they said for us we are going to continue using the MCH Working Group and they [GFF] allowed. But as we went along, we realized that the MCH Working Group was not inclusive for everybody, so we had to structure it and create the extended MCH Working Group structure which is now used as a country platform (Respondent 7)

There was no consensus among the respondents about the inclusion of some stakeholder groups, such as young people/adolescents, cultural and faith leaders, other government Ministries, the elderly and community members. While some respondents gave examples of missing actors who they felt were not involved in the IC development process, e.g. academics, district-level actors, private sector and women’s groups; other respondents indicated that these groups were included. Additionally, the government and GFF actors emphasised that CSOs were included in the technical working groups that developed the investment case. On the contrary, CSO respondents indicated that they had not been involved, particularly from the start, and only heard about it from their global networks.

Throughout the development of the IC, the technical working group held regular meetings at different stages to review evidence and discuss what to prioritise and include. The process of developing the IC required a blend of both technical expertise and financial support, provided by the GFF and UN partners. Certain members of the group also helped to write sections of the policy document.

The Ministry of Health led the development of the Investment Case, but there were many stakeholders. There were UN agencies including UNICEF, WHO, UNFPA, district health teams, implementing partners, and civil society organizations led by World Vision. (Respondent 5)

Unlike the IC, where a significant portion of how it was developed and who was involved was detailed in the document, the PAD’s development was not similarly described in the document. We relied heavily on interviewees to better understand the process of PAD development and finalisation. According to respondents, the PAD was negotiated through a separate structure convened by the Ministry of Finance, Planning and Economic Development (MoFPED). This divergence was necessitated by the nature of the PAD, which involved negotiating an International Development Association credit that was merged with GFF funds. Technocrats from MoFPED worked with MoH and the National Planning Authority in developing the PAD. Following World Bank board approval, MoFPED also presented the request for credit to the parliament in November 2016 as part of the legal process for receiving a World Bank loan [22].

The reason for the expedited parliamentary approval was partly linked to the proactive engagement of members of parliament, which happened well before the official request reached parliament through their respective parliamentary committees.

Parliament is an important actor; it must approve the loan. We engaged parliament proactively. There are parliamentary committees that we engage during and even before tabling the bill to the House. We tried to make them understand what the credit was about but also allowed them to contribute their insights in advance. With this in place, it was easy to get parliamentary approval. (respondent 3)

Both the IC and PAD engaged diverse actors with varied interests. For instance, the IC development involved technical RMNCAH experts and the use of evidence was prioritised. The PAD process involved mostly non-technical actors introducing new dimensions of interest, key among whom were the Members of Parliament. Consequently, there was a higher degree of contention between actors in the PAD development. The concerns of Members of Parliament were that certain areas like drug stockouts, medical equipment and facility infrastructure had not been given due consideration. Accordingly, members of Parliament insisted on shifting some PAD allocations to more ‘visible’ items that could earn them political clout.

There was politics; when the proposal went to parliament, people thought these are the ways of getting capital, because when you build a health center, it is going to help the ‘wanainchi’[common man](Respondent 9)

However, one respondent pointed out that the GFF grant funds in the PAD were preordained by GFF to be exclusively given for results based financing and civil registration and vital statistics. Consequently, there was no room for negotiation to move some of the funds to other areas through parliamentary or other in-country processes.

Despite mixed reporting about CSOs’ engagement in the IC process, CSOs played an instrumental role in influencing the content of the PAD. For instance, upon hearing about the GFF from global counterparts, CSOs strategically engaged in the PAD development process through their existing parliamentary advocacy channels and identified critical areas missing from the project, such as family planning, which was then added.

We were not engaged in the development of the Investment case. But we learnt about the PAD from our connections outside Uganda and we quickly acted. So, we made engagements and advocacy with the Ministry of Health and the Parliament of Uganda to make sure that the PAD is aligned and includes indicators that got the whole spectrum of family planning and reproductive health which was not well formed … we got that indicator formed in the PAD (Respondent 8)

Supplementary file 5 includes a mapping of the actors, their roles and interests in the development of GFF documents.

### Theme three: (mis)understanding of the GFF

There was some reported dissemination of the GFF purpose and plans to the various stakeholders when it started. Nonetheless, most respondents had no idea why the GFF was established or how it started globally and in Uganda. With the exception of a few senior officials who were directly involved in the process, there was even less knowledge available on the PAD. Mirroring the lack of clarity about how GFF started globally or in-country, few people understood what the GFF money was meant to achieve despite having been briefed about the GFF by MOH, World Bank and other partners.

One respondent shared that there was a belief amongst civil society at the time the GFF was introduced that it was ‘bringing in a chunk of money’ for RMNCAH that would be distributed amongst different stakeholders, not only government. This is however not the model of GFF and respondents explained that subsequently, some people were dissatisfied and suspicious.

They said they are going to bring a lot of money to the country, yet the money was very little … Secondly, they said GFF was going to establish all structures at country level, but they didn’t know that the funds were going through the [World] bank. (Respondent 7)

Stakeholder engagement and participation throughout the process was another misunderstood expectation. After multiple consultations during the IC development process, MoH proceeded on the project (PAD) with less partner engagement, only giving periodic reports on progress. Some groups, including donors, felt excluded from the implementation process, demonstrating this misunderstanding about what the PAD represented as it links to the GFF investment case.

Why donors felt left out of the GFF and World Bank RBF project was because they knew it was premised on the RMNCAH investment case, but they were not necessarily invited to the table to discuss progress there. But at the joint meetings progress was articulated (Respondent 2)

## Discussion

This study describes the introduction of GFF, a global health initiative, into Uganda’s national RMNCAH agenda. Our findings show that the initiative may be termed as largely ‘country-led’, since it leveraged using national structures led by government. However, the process was also significantly influenced by global actors. There were numerous opportunities for actors to participate in policy development and influence the processes involved. As the central coordination team, the MoH had to deal with numerous actors, each with their own set of interests. The main point of contention was between technical priorities and political motivations, in addition to a lack of consensus between the respondents about which groups were engaged or excluded from the IC development processes.

In Uganda, the development and approval of the GFF documents was remarkably quick in comparison to the norm for other policies, which typically take more than a year [25]. Many reasons contributed to the quick development. First, the IC was being adopted from a well-established Promise Renewed which had undergone a rigorous development process. The goal was to identify the most critical priorities for investment among those proposed in the Promise Renewed. Additionally, the coordinators at MoH demonstrated expertise in handling the processes. For example, they made an effort to proactively engage key stakeholders, like parliament, getting their buy-in and thus expediting the entire process of parliamentary approval, which would ordinarily have encountered delays.

Although the rapid development of policy was commendable, it was not without challenges, including limited engagement of all actors. Even though some CSOs are part of existing governance structures, CSOs are a diverse group of actors and achieving their full representation requires time and extensive mobilisation. With the rapid time frame, such mobilisation was not feasible. Indeed, time has been noted to be an indispensable factor influencing meaningful engagement [11]. Importantly, the role of CSOs in global health initiatives is imperative. Intentional effort is needed to ensure adequate consultation and buy-in [26].

Local actors, such as communities, women’s groups and traditional leaders who were not directly engaged in the GFF process found themselves as mere recipients of policies enacted by various elite groups, which essentially meant that the GFF maintained the status quo [28]. The GFF policy documents were primarily developed by technocrats and senior management at the MoH with support from development partners and some academia. This approach to policy development has been characterised as ‘elite-led policy’ as described by Mukuru and others [6]. The involvement of members of the parliament in the PAD process introduced another group of elites, though they were motivated by political interests rather than technical interests.

For many years CSOs have strategically been part of the Ugandan parliamentary committees and play a vital role in influencing priorities at that level [11]. Our study reflects this norm, showing that the local CSOs are indeed powerful national policy influencers who could indirectly set priorities through asserting pressure on politicians, via their advocacy platforms. The lobbying of Parliament by CSOs reportedly arose only after receiving information from global actors. This reflects a strategy often used in countries to navigate donor influences called ‘shadow diplomacy,’ where ‘informal networks and channels of influence run parallel to, but are not recognised as part of formal diplomacy’ [29]. This scenario also underscores a trend where global actors (donors) are increasingly interested in working with local CSOs and are in tandem increasing their financial support to these organisations [31]. The close collaboration between CSOs and global actors has led to a development where the latter leverage CSOs to advance their interests [27,30]. This dynamic reduces CSO advocacy efforts to a tokenistic endeavour, serving as a platform for promoting the agenda for donors [34]. It is important to recognise the significant value of CSOs as strategic local allies for global actors.

Despite the interest in civil society’s involvement in the GFF, there was a lack of direct funding from the GFF to CSOs. This contrasted the approach of other global health initiatives and donors in Uganda [6]. Our findings showed that the lack of cash flow from the GFF to stakeholders, particularly CSOs, resulted in widespread dissatisfaction and misunderstanding of the initiative by civil society actors. This misunderstanding resulted in a multitude of contradictions and unwarranted opposition directed at the MoH and GFF. A notable example is a prominent CSO that expressed scepticism toward the GFF model [13,14]. As a consequence, the goal of designing a country-led model faced some struggles and could have potentially hurt implementation, particularly in terms of cultivating collaboration with diverse stakeholders [16].

The study brings to light an aspect that warrants further scrutiny: the use of general terminologies like ‘country-led’ or ‘national priorities’. In the country, global actors, including the GFF and other donors who endorsed the GFF model, actively worked with local ‘elites,’ that is, local technical people and politicians through providing technical and financial support, thereby securing local legitimacy for a globally conceived plan. Despite meeting the criteria of what may be labelled as ‘country-led,’ it raises the question of whether it truly represents citizens’ priorities. One review indicated examples of where global health initiatives leverage their financial and technical affluence to determine country priorities [32]. Although the GFF undeniably bore the stamp of being ‘government-led’ and indeed used established government structures, this may not equate to truly representing local RMNCAH priorities [31,33].

Nevertheless, learning and improvements in the development of GFF policy documents are ongoing and the whole process has been versatile [16]. The Ugandan government released its second Investment Case in 2022 with some notable changes, including strengthened engagement with the private sector [23]. The GFF policy-related processes have evolved since its initiation, with a broader national country platform for Maternal and Child Health that is more inclusive of CSO members, which meets quarterly (June 2023 correspondence between the GFF, MoH officials and the authors).

In terms of policy implications, the MoH must address local stakeholders’ understanding of the GFF, to ensure accurate expectations going forward and avoid breeding suspicion and allegations. This can also increase buy-in and guide stakeholders’ participation in the development and implementation of the Investment Case. This can be done in various ways, including sensitisation by MoH and GFF of various stakeholder categories on the GFF model and how it operates. The MoH should also increase sensitisation and popularisation of the sharpened plan nationally and sub-nationally. There should be clear communication on what a new funding model entails, its purpose and how it shall be managed.

Ensuring a more inclusive planning process is important and while more categories of stakeholders have been incorporated, more still needs to be done. We specifically recommend that the MoH, as the coordinating institution, deliberately involves sub-national district leaders right from conceptualisation so that eventually implementation is easier and more effective. Additionally, improved collaboration with other government institutions that are interlinked, or that impact health service delivery is neededfor instance, the Ministry of Local Government. The private sector is a critical actor that was previously not involved in the IC and yet plays a significant role in health service delivery in the country, providing close to fifty percent of health care. CSOs also need to strengthen sub-national engagement and representativeness in such national processes, so that they are always prepared for engagements as opportunities arise.

It is imperative to identify ways in which the GFF process can integrate more local voices in order to align effectively with the local RMNCH priorities of Uganda. Additionally, the technical assistance provided by the GFF should be leveraged to build local capacity to ensure future sustainability and less need for reliance on external actors. It is also important to keep track of global health initiatives and processes, especially where these have direct implications at national and sub-national levels so that one can know when, where how and if to engage.

### Strengths and limitations

The study was limited by the fact that the processes under study happened some years ago and respondents may have had difficulty recalling certain aspects of what occurred. Additionally, not all stakeholders involved in the process were interviewed for this study because they had moved from those positions and/or were unavailable for other reasons. For example, only half of the key informants contacted by us from donor organisations agreed to participate in the interviews. This could have resulted in biased perspectives, with donors providing fewer insights than other actors. We addressed this by holding validation meetings where donor organisations were invited to confirm the findings of the research. They did so by sharing additional information, which were considered in relation to the preliminary findings and increased the study’s comprehensiveness.

Also, it was outside the remit and capacity of this study to expand the data collection to include participatory research approaches with affected communities, notably those absent from the process. Further research with these communities on their perspectives and experiences of the policy processes related to RMNCAH priority setting, funding mechanisms and implementation follow-up would be recommended. Furthermore, because our primary focus was on the GFF’s inception phase, we did not investigate more recent advancements in the country’s GFF policy formulation procedures. Nonetheless, this study interviewed a diversity of actors who were involved and triangulated data sources, considering the data from the existing literature, the desk review, and key informant interviews. The key informants were particularly chosen to ensure a diversity of views across different groups that were involved in the processes of developing the IC and PAD.

The content analysis of policy documents was limited by what documents were available publicly or provided by the stakeholders. For this study, we only assessed the content of these documents and not the quality of their content. Future research may want to consider the validity of the contentfor example, the evidence-based solutions presented.

The study was conducted by an interdisciplinary team with experience both in Uganda and internationally, as part of a broader series that includes other country case studies from Burkina Faso, Tanzania, and Mozambique, in addition to content analyses of GFF documents. This collaboration met frequently to reflect on the various studies, pooling together their breadth and depth of knowledge. In order to increase the credibility and validity of study findings, three validation meetings were held in May and June 2023 with representatives of MoH, the GFF secretariat, and CSOs. Participants reviewed and provided feedback on findings during these meetings and helped to validate, supplement, and clarify the information gathered through interviews.

## Conclusions

Policy development for the GFF policy documents in Uganda was government-led, with a multiplicity of actors involved. However, the exclusion of some important groups initially led to mistrust and confusion around the GFF funding mechanism. There were also challenges related to balancing the various actor interests and priorities and navigating the rapid policy development process. Our findings suggest that implementing a globally developed agenda at the country level is a political and technical process, requiring early engagement with different key country stakeholders to ensure they understand it and have their buy-in, particularly if certain elite interests whether international or national are to be balanced.

## Supplementary Material

GHA special series GFF Priorities Processes and Investments.docx

Supplementary files.docx

## Data Availability

The datasets used and/or analyzed in this study are available from the corresponding author on reasonable request. This paper is part of a *Global Health Action* Special Series. A more detailed explanation of the full study and sub-analyses will be available in Volume 17-01.
