# Supplementary material for: Policy analysis of the global financing facility in Uganda
Source: Glob Health Action. 2024 Jun 19;17(1):2336310. doi: 10.1080/16549716.2024.2336310 (PMC11188944; doi:10.1080/16549716.2024.2336310)
Supplement: GHA special series GFF Priorities Processes and Investments.docx [file ZGHA_A_2336310_SM4809.docx]

GLOBAL FINANCING FACILITY FOR WOMEN, CHILDREN, AND ADOLESCENTS: EXAMINING NATIONAL PRIORITIES, PROCESSES, AND INVESTMENTS

Special Series GHA concept note

# Background

The Global Financing Facility (GFF) hosted by the World Bank, was launched in 2015 with the intent to support countries with the largest burden of reproductive, maternal, newborn, child, adolescent health and nutrition (RMNCAH-N) and accelerate progress to meet Sustainable Development Goals by 2030.^1^ The GFF offers an opportunity to close financing gaps, particularly in the 36 countries being supported, aiming to mobilize more than US$57 billion from 2015 to 2030 through domestic resources, new external support and improved coordination of existing assistance. To date, limited independent published analyses exists examining GFF commitments and investments.^2^ A Special Series in *Global Health Action (GHA)* is planned for 2023 to bring together independent, multi-country, policy analyses assessing the GFF in low and middle-income countries.

# Special Series aim and objectives

This Special Series aims bring together analyses from multiple studies that assess GFF related policy content, processes and power from a range of countries where the global health intiative is operating. Publishing these analyses in a Special Series will enable broader contextualisation of the emerging findings and enable more nationally-led, evidence-based policymaking in the 36 GFF countries. It will also help inform national and multilateral stakeholders in promoting global accountability in investments for the most vulnerable populations that are the stated priority of GFF.

Specific objectives include:

- To undertake policy content analyses to systematically assess how the GFF-related country planning documents address vulnerable populations, including:
  - Adolescents with sub-analysis on gender
  - Postpartum women, stillbirths, newborns with sub-analysis on quality
  - Community
- To use case study methodology to explore the political processes and power involved in developing and implementing the GFF country planning documents at national level.
- To map the GFF investment in countries with the need in terms of burden, likely impact and stated priorities by national governments, the GFF, and World Bank.

# Teams

The work is being led by the Countdown to 2030 Health Policy and Systems group, based at the University of the Western Cape, in partnership with NEST360 colleagues at the London School of Hygiene & Tropical Medicine (LSHTM) and Makerere University. The individual research articles were developed with a group of independent, multi-disciplinary researchers in Africa. Now called the “Countdown GFF policy analysis collaboration,” this group has organically grown with intentional efforts to facilitate South-South knowledge exchange, shared learning and enable equitable partnerships. The Editorial team for the GHA Special Series includes the Guest Editors (Professor Asha George, Professor Joy Lawn, and Professor Peter Waiswa) as well as Drs Mary Kinney and Meghan Kumar.

# Special Series papers

The Special Series is proposed currently as 13 papers (10 research articles, two commentaries and one editorial). Papers will include authorship from the different teams engaged in the data collection, analysis and write up based on authorship principles. GHA allows for rolling submissions; thus papers will be submitted as ready.

Commentaries

1. GFF at 6 years: taking stock
2. Southern/Country-led mechanisms as way forward

Articles

1. Systematic, multi-country analyses of content of GFF ICs and PADs and country experiences
2. Case study: Tanzania
3. Case Study: Burkina Faso
4. Case Study: Mozambique
5. Case Study: Uganda
6. Vulnerable groups: Do stillbirths, and maternal-newborn outcomes count in GFF ICs and PADs?
7. Vulnerable groups: Has priority for adolescent health continued across ICs and PADs?
8. How is gender addressed across GFF ICs and PAD?
9. How is community health addressed across GFF ICs and PADs?
10. Does quality count across GFF ICs and PADs?

Editorial

1. Closing: aligning stated priorities and financings – where do we go from here?

For more details, contact the project coordinator: Mary Kinney [mkinney@uwc.ac.za](mailto:mkinney@uwc.ac.za)
