# Supplementary material for: Policy analysis of the global financing facility in Uganda
Source: Glob Health Action. 2024 Jun 19;17(1):2336310. doi: 10.1080/16549716.2024.2336310 (PMC11188944; doi:10.1080/16549716.2024.2336310)
Supplement: Supplementary files.docx [file ZGHA_A_2336310_SM4808.docx]

**Supplementary files: Turning the Global Financing Facility agenda into a nationally owned plan: A policy analysis in Uganda**

Contents

[Supplementary file 1: Country context 2](#_Toc146870370)

[Supplementary file 2: Data collection tools 3](#_Toc146870371)

[Supplementary file 3: Policy analysis 4](#_Toc146870372)

[Supplementary file 4: Equitable partnership background and statement 5](#_Toc146870373)

[Supplementary file 5: Additional results 7](#_Toc146870374)

# **Supplementary file 1: Country context**

Table S1.1: Key indicators for Uganda (source GFF data portal <https://data.gffportal.org/country/uganda>)

| **Indicator** | Estimate | Year |
| --- | --- | --- |
| **DEMOGRAPHICS** |  |  |
| Population | 47,250,000 | 2021 |
| Population that are adolescent (10-19 yrs) | 11,920,000 | 2021 |
| Total fertility rate (births per woman) | 4.5 | 2021 |
| Maternal deaths | 6000 | 2021 |
| Under-5 child deaths | 69,025 | 2021 |
| Neonatal deaths as % of all U5 deaths | 46 | 2021 |
| Stillbirths rate (per 1000 total births) | 15 | 2021 |
| **COVERAGE (%)** |  |  |
| Demand for FP satisfied by modern methods | 55% | 2019 |
| Antenatal care (4+) | 57% | 2019 |
| Skilled attendant at delivery | 76% | 2019 |
| Postnatal care for newborns | 54% | 2019 |
| **CONTEXT** |  |  |
| Health worker density (doctors) per 10,000 population | 1.54 | 2020 |
| Health worker density (nurses, midwives) per 10,000 population | 16.37 | 2020 |
| **FINANCING** |  |  |
| General government expenditure on health as % of total government expenditure | 6% | 2020 |
| Out of pocket expenditure as % of total expenditure on health | 37% | 2020 |
| Investment case (total, USD million) | 3% | 2020 |
| GDP per capita (current US$) 2010−2019 | 3% | 2020 |

**Narrative summary of Uganda’s context during the time period of study**

Uganda was in the middle of presidential and parliamentary elections in February 2016, when the GFF was introduced and IC and PAD were developed. Despite the elections, there were few changes in political leadership, with the president remaining in office and his ruling party gaining a sizable number of parliamentary seats. This electoral outcome resulted in the same ruling party maintaining significant influence over various national-level priorities and decisions. Furthermore, the policy is scrutinized by both the parliament and the cabinet, to ensure that it serves the government's aspirations. Stakeholder participation is integrated throughout all processes [1].

The National Development Plan (NDP) Strategy typically documents the government's aspirations and strategic implementation plans. The NDPs are intended to be the government's primary strategic plan, upon which sectoral plans and the national fiscal strategy will be based. Around the same time, the country transitioned from National Development Plan 1 (2010/11-2014/15) to National Development Plan 2 (2015/16-2019/20) ([2][3]. The NDP II was established in the midst of a reduction in the government's budget allocation to health, which stood at 8.6% of the total budget in 2014/15. Despite this reduction, health was explicitly identified as a priority area for human capital development. The main infrastructure projects relied on anticipated external borrowing [4].

Between 2010-2014, the country’s economic growth averaged 5.5%, with a fluctuating fiscal budget deficit that was 5.0% in 2013/14, with total domestic debt stock at 10.4% of GDP and 15.8% for external debt (REF:NDP II). Uganda’s debt stock reportedly increased from $5.5. billion to $9.8 from the year 2000 to July 2017 respectively [5] and this is part of the environment in which the IC and PAD were developed. Between June 2015 and June 2016, Uganda’s Ministry of Finance indicated low potential debt distress for the country, with an increased external debt from $4.4 billion to $5.2 billion that was considered sustainable in the medium to long term, despite some vulnerabilities [4]. Multilateral lenders were owed 77% of the external debt at the time.

#

# **Supplementary file 2: Data collection tools**

**Interview Guide**

*The first three questions are about the GFF in general; we would like to know what you know about it, its objectives and why it was set up.*

1. Could you please share what you think the GFF mechanism is about, and why was it created, and what are its objectives?

- Probe: What are the expected benefits of the arrangement

1. Please explain how the GFF started in Uganda?

- *Probe*: Explain how the PAD was developed.
- *Probe*: Explain how the IC was developed?
- *Other probes for each depending on key informant’s background*
  1. How did you first hear about it?
  2. When did it start?
  3. How did development begin? What was done? Was it a consensus fluid process, or were there challenges, things to improve?

1. Can you please describe who were core people involved in the development of the documents?

- Probe: For the key actors, how were they able to drive the process and what challenges did they face?
- Probe: Which group of actors (Ministry of Health, World Bank, Ministry of Finance, others) had the most influence over the content of the IC and PAD? What kind of influence did they have; how and why
- Probe: Do you think there were any other actors which influenced the development process and priorities of the IC and PAD? If so, please explain which actors and how they influenced the process?
- Probe: In your opinion, where important actors absent from the process. If so, explain why.
- Probe: In your opinion, do you think any actors opposed the GFF processes? Why??

1. Can you describe your position and what your role was in to the process developing RMNCAH Investment case and Program appraisal documents?

- Probe: Were you involved in IC, PAD, or both
- Probe: Do you think you were sufficiently involved? If yes, how? If not, why?

Note to interviewer: you want to find out who else was involved and their roles - who was leading the process, who was participating, who not engaged. What you ask will depend on the participant being interviewed.

1. What were the key inputs and events supporting the development of the GFF investment case and PAD?

- Probe: explain if there were special data analyses conducted or frameworks used, what did this entail?
- Probe: explain if there was there stakeholder consultation, what was this like? who was involved or not? how smooth?
- Probe: explain if there was there multi-sectoral engagement, what was this like?

1. How were priorities and investments (budgets) determined in the IC and PAD?
   - Probe: How does the GFF programme relate to / correspond with MOH’s strategic and planning documents? other programmes including in other sectors?
   - Probe: Do you think any key issues were being left behind? How and why?
   - Probe: Did any special advocacy groups engage and influence the content of the documents?
2. Did the World Bank or GFF Secretariat provide any guidance (including but not limited to good practices) on how to develop the investment case or PAD?
   - 1. If yes: can you share this guidance with us?
     2. If yes: To what extent would you say these good practices were followed and what facilitated or hindered this.
3. In your opinion, what were the key milestones or activities relating to the GFF document development process?
   - Probe: Was there an initial meeting with all stakeholders?
4. Can you share any specific challenges experienced in Uganda during the development of the IC and PAD?

- Probe for contextual factors that may have influenced the process (e.g. Political; Economic; Historical, health system change, external donors) and find out how it affected the development process.

1. .Do you think that this GFF mechanism has led to more or better-aligned resources for health? Can you give some examples?
   - Probe: How did other donors react to the introduction of the GFF mechanism?
   - Probe: How did domestic resource allocation change/ increase? why or why not?
   - Probe: Was there any engagement from the private sector? If yes, how, what, why

*Summary - perceived implementation by those involved in the development of the documents*

1. In your opinion, has the implementation of the GFF mechanism in Uganda kept all its promises? Explain your answer
   - Probe: What were the contributing factors (barriers or enablers) in your opinion?
   - Probe: Based on your experience of developing the last one, what do you think needs to be improved as the country develops its next IC and PAD? Why do you think so.

# **Supplementary file 3: Policy analysis**

Figure 3.1: Framework for understanding the GFF country document development and implementation processes


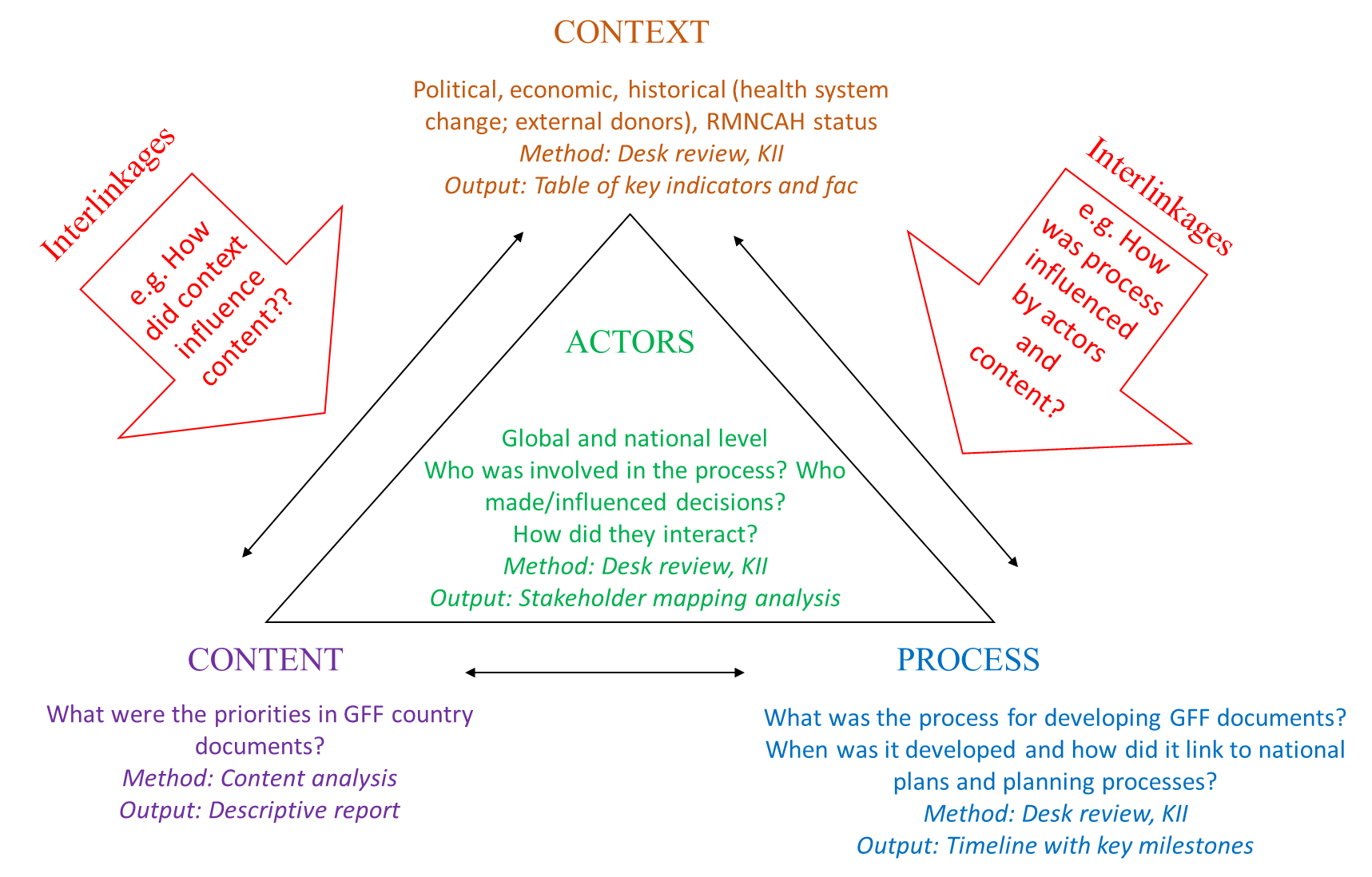


Source: Reference paper 3

# **Supplementary file 4: Equitable partnership background and statement**

The Countdown Global Financing Facility (GFF) policy analysis collaboration co-developed a “principles of an equitable partnership” document. The purpose was to provide background on the GFF policy analysis collaboration, clarify roles and responsibilities, and document ways of working, including data governance and authorship principles to strive for equitable partnership in the collaboration.

**Brief history of collaborative**

The Countdown to 2030 Health Policy and Systems group, based at the University of the Western Cape (UWC), initiated this work in an effort to build on their previous content analysis of GFF documents. UWC and the NEST360 project at the London School of Hygiene and Tropical Medicine (LSHTM) started to collaborate in Q4 2020 with an aim to focus on the content of maternal and newborn health (MNH) in these documents. RESADE joined the team in mid-2021 initially to support on the Francophone documents for the adolescent health and MNH content analyses. The concept of expanding this work to include country studies emerged from these partnerships and the results from the content analyses. UWC and LSHTM identified potential partner organizations in other GFF countries that had been involved in the GFF processes or independent assessments of the GFF processes and with whom teams had worked with previously. The organizations initially approached agreed to collaborate on this work, N’weti (Mozambique), Makerere University (Uganda) along with RESADE (Burkina Faso) and discussions began in Q2 2022. Through NEST360, Ifakara Health Institute and the GFF Tanzania office also agreed to join this partnership in Q3 2022. To support the work, the UWC sub-contracted these teams in Burkina Faso, Mozambique and Uganda through the Countdown to 2030 project, providing some resources to support people tiime. Similarly, LSHTM through NEST360 is supporting the time of their team members and those working on the Tanzania study. Table 1 provides a list of the partner organizations involved in this work and specific team members engaged. The team expanded organically overtime.

**Table 1: Organizations and teams engaged in this partnership**

| **Organizations** | **Team** |
| --- | --- |
| RESADE | Joël Kiendrébéogo, Yamba Kafando, Issa Kaboré and Orokia Sory |
| N’weti | Andes Chivangue, Denise Namburete, |
| Ifakara Health Institute/NEST | Donat Shamba, Jitihada Baraka |
| World Bank/GFF | Georgina Msemo |
| Makerere University | Phillip Wanduru, Doris Kwesiga, Peter Waiswa |
| LSHTM/NEST | Meghan Kumar, Joy Lawn, Rosie Steege |
| UWC | Asha George, Mary Kinney, Ulla Walmisley |

**Principles for this collaboration include:**

- Inclusivity of teams and individual members within each team in weekly meetings and email correspondences, including rotating chairs for the weekly meetings
- Acknowledgement that teams come with their different histories and formal or informal hierarchies
- Co-design and co-development of study concept, data collection tools, data analysis approach, synthesis of results (paper structure), and knowledge translation plans
- Open sharing of experience and findings of data through weekly meetings and workshops
- Open sharing of information, summary data tables and paper drafts on a google drive only accessible to those who are part of the collaboration. Teams may access each other’s files for review as examples for learning; teams may share files or not on the google drive, the decision rests with each team to upload documents on the google drive.
- Agreement on data ownership, particularly by country teams.
- Agreement on processes of local data validation and feedback ahead of sharing results with global audiences.

**Areas of collaboration**

- For the case study research, the collaboration worked together on the conceptualization and general approach of the study, including co-designing of the data collection tools, analysis framework, and analysis tools.
- For the content analysis research, the collaboration worked together to identify documents for inclusion, with input from the GFF secretariat, identify a systematic approach using the READ technic (ref), and advanced the conceptual framework, called the Ms Framework, used to conduct the analysis. Teams applied the framework in different ways depending on the topic of study.

*The full partnership agreement is an internal document. Please contact the corresponding author if you would like more information.*

# **Supplementary file 5: Additional results**

*Table S5.2: Comparing contents of the related RMNCAH policies in Uganda related to GFF*

|  | **Promise Renewed (2013)** | **Investment Case (April 2016)** | **Project Appraisal Document (June 2016)** |
| --- | --- | --- | --- |
| Brief description of document / high level overview | - A national level guide for improving reproductive health services in Uganda holistically | - A national level guide for improving reproductive health services in Uganda holistically | - A WB project document that focuses on improving health service delivery – prioritizing governance, accountability, and service delivery. |
| Focus areas: goals | - Maternal, newborn, infant, and child health services. | - Maternal, newborn, infant and child health, plus adolescent health. | - The Project Development Objectives (PDOs) are to: (a) improve utilization of essential health services with a focus on reproductive, maternal, newborn, child and adolescent health services in target districts; and (b) scale-up birth and death registration services |
| Strategy | - Five strategic shifts: i) high-impact interventions ii) high burden/ underserved populations iii) sequencing iv) multi- sectoral v) accountability | - Five strategic shifts: i) high-impact interventions ii) high burden/ underserved populations iii) sequencing iv) multi- sectoral v) accountability | - Strategic shifts not mentioned |
| Cost | - USD 681 million USD | IC costed in three scenarios over 5 years:  1. Current coverage maintained = US$ 1.6B  2. Rapid scale up of prioritized core and expanded packages = US$ 1.92B   - 3. Expanded package delivered nationwide = US$ 2.2B | USD 140 million   - Component 1: Results-Based Financing for Primary Health Care Services USD 68M - Component 2: Strengthen Health Systems to Deliver RMNCAH Services USD 54.5 M - Component 3: Strengthen Capacity to Scale-up Delivery of Births and Deaths Registration Services USD 10 M - Component 4: Enhance Institutional Capacity to Manage Project Supported Activities USD 7.50 M |
| Mapping resources | - No | - Yes, mapped existing resources | - Not needed |
| Distribution of funds | - Non-specific, funds allocated in broad packages | Non-specific, funds allocated in broad packages like community package, HC III package, HCIV package and above | Specific/clear   - Component 1: Results-Based Financing for Primary Health Care Services USD 68M - Component 2: Strengthen Health Systems to Deliver RMNCAH Services USD 54.5 M - Component 3: Strengthen Capacity to Scale-up Delivery of Births and Deaths Registration Services USD 10 M - Component 4: Enhance Institutional Capacity to Manage Project Supported Activities USD 7.50 M |
| Length /duration of investment | 2013 to 2017 | 5 years (2016/17-2019/20) | 5 years (2017-2021) |

| Actors | | Type of power | How they influenced the IC | How they influenced the PAD | Their interests |
| --- | --- | --- | --- | --- | --- |
| Ministry of Health | | - Political mandate - Technical knowledge | Led development of IC | Central in development of PAD, working with world bank and GFF | - Mandated with delivery of health services in Uganda |
| Parliament | | - Political mandate |  | Moved funds for infrastructure and HR | - Directly accountable to population - Gaining political currency |
| MoFPED | | - Political mandate - Technical knowledge |  | Supported in development of PAD, working with world bank and GFF | - Overseeing financing of health care services |
| NPA | | - Technical knowledge |  | Central in development of PAD, working with world bank and GFF | - Overseeing planning for country |
| UNICEF  UNFPA | | - Financial power   Technical knowledge | - Funded development of IC - Provided technical assistance. - Participated in TWG meetings when developing IC |  | - Supporting countries to achieve global RMNCAH goals |
| World bank* | | - Financial power - Technical knowledge | - Participated in TWG meetings when developing IC | - Led development of PAD - Provided the funds | - Supporting countries to achieve global RMNCAH goals |
| GFF* | | - Financial power - Technical knowledge | - Participated in TWG meetings when developing IC | - Supported development of PAD - Provided funds | - Supporting countries to achieve global RMNCAH goals |
| Other donors USAID* | | - Financial power   Technical knowledge | - Participated in TWG meetings when developing IC |  | - Supporting countries to achieve global RMNCAH goals |
| WHO* | | - Political mandate   Technical knowledge | - Participated in TWG meetings when developing IC |  | - Supporting countries to achieve global RMNCAH goals |
| CSOs | | - Capacity to influence actors with political mandate.   Technical knowledge | - Participated in TWG meetings when developing IC |  | - Supporting countries to achieve global RMNCAH goals. |
| Academia | | Technical knowledge | - Participated in TWG meetings when developing IC - They were consultants in evidence gathering |  | - Supporting country to achieve RMNCAH goals |
|  | **Consolidated power | | | | |

*Table S5.2: Mapping the actors, their roles, and interests in development of IC and PAD*

References

[1] U. Government, “A Guide to Policy Development & Management in Uganda,” 2013. [Online]. Available: http://regulatoryreform.com/wp-content/uploads/2016/09/Uganda-Revised-Guide-to-Policy-Development-Mgt-2013.pdf

[2] M. of Finance, “SECOND NATIONAL DEVELOPMENT PLAN 2015/16 – 2019/20 (NDPII),” 2015. [Online]. Available: http://npa.go.ug/wp-content/uploads/NDPII-Final.pdf

[3] NPA, “NATIONAL DEVELOPMENT PLAN VISION: A TRANSFORMED UGANDAN SOCIETY FROM A PEASANT TO A MODERN AND PROSPEROUS COUNTRY WITHIN 30 YEARS THEME: GROWTH, EMPLOYMENT AND SOCIO-ECONOMIC TRANSFORMATION FOR PROSPERITY (2010/11 - 2014/15),” 2010. [Online]. Available: https://planipolis.iiep.unesco.org/sites/default/files/ressources/uganda_ndp_april_2010.pdf

[4] P. A. E. D. MINISTRY OF FINANCE, “DEBT SUSTAINABILITY ANALYSIS REPORT 2016/17,” 2017. [Online]. Available: https://mepd.finance.go.ug/documents/DSA/DSA-FY201617.pdf

[5] R. Ssempala, K. Ssebulime, and E. Twinoburyo, “Uganda’s experience with debt and economic growth: an empirical analysis of the effect of public debt on economic growth—1980–2016,” *J. Econ. Struct.*, vol. 9, no. 1, p. 48, 2020, doi: 10.1186/s40008-020-00224-2.
